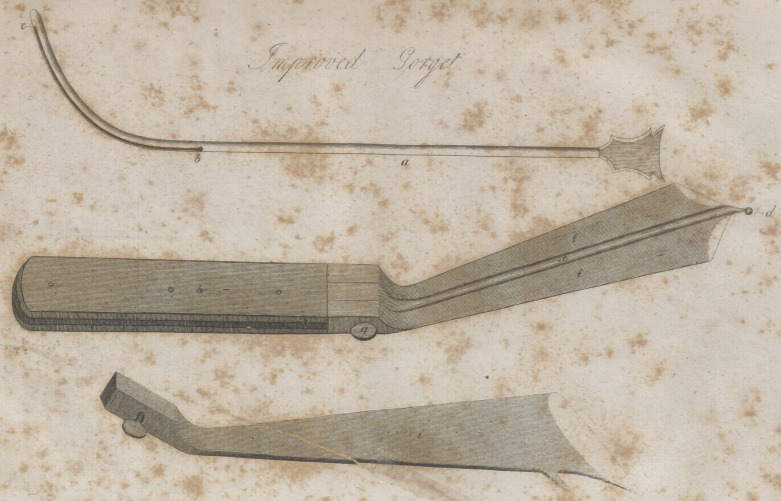# Lithotomy

**Published:** 1835

**Authors:** Joseph N. McDowell

**Affiliations:** Professor of Special and Surgical Anatomy in Cincinnati College; Cincinnati


### Art. IV.—Lithotomy.

*Desultory Observations on Lithotomy, with Remarks on some Defects in the Common Gorget.* By Joseph N. McDowell, M. D., Professor of Special and Surgical Anatomy in Cincinnati College.

The operation for stone in the bladder has from the infancy of the profession of surgery, been viewed as one of the most formidable to which the human frame can be subjected, and therefore, as one of the highest achievements of the practitioner in the art.

The reason the operation has been thus considered, and approached with so much terror, by both patient and surgeon, must be obvious:—the parts through which the instruments employed are to pass, being both delicate and complicated, and the bill of mortality probably greater in this than in any other operation.

But the history of the operation of lithotomy will most conclusively teach us, that the want of success has been justly attributable to the want of proper instruments, or the malconstruction of those employed, rather than to a deficient knowledge of the parts concerned in the operation. Although these parts were described with much accuracy by Celsus, his history of the operation and the result, conveys the idea that it was rude and unsuccessful, because of the surgeon's being unable to use with any certainty his instruments. The plan first devised, and termed the Celsean operation, (though originating in the school of Alexandria several hundred years before,) was the simple incision on the stone, when pressed against the perinæum, by the introduction of the two first fingers of the left hand into the rectum, and grasping the stone, and forcing it down. The want of success was, doubtless, owing to the great uncertainty as to what parts the knife would pass through, for much must have depended on the form and size of the stone, as well as the direction towards the perinæum, given it by the fingers. Thus parts were often injured, the restoration of which could not readily be effected, and cutting for the stone was almost as fatal as the malady itself.

The introduction of this operation by the most learned and classic of Roman authorities, tended much to its perpetuation in Europe, and, although rude, it was practiced for more than thirteen hundred years.

But the writings of Hippocrates, which were brought into Europe at a much later period, and the implicit confidence extended to his aphorisms, gave rise to another mode of extracting the stone. The proposition was, to remove the calculus by dilating the urethra, inasmuch as the father of medicine had declared, that wounds in the membranous parts of the body were mortal, and for this purpose a variety of instruments were constructed, the number and peculiarities of which have not been surpassed by those employed in any operation either before or since. The remarkable difference in the number of instruments used in this mode of operating, and that advised by Celsus has given the name of the apparatus major to the one, and that of apparatus minor to the other. In the latter, only the knife and hook are used, while in the former a small volume would scarcely suffice for their description.

The absurdity of this operation, soon, however, brought it measurably into disuse; for while its advocates contended that the urethra should not be wounded with the knife, but that it was susceptible of a dilatation sufficient for the passage of any stone, they not only cut into the membranous portion of the urethra, but by the rudeness of their dilators, lacerated the prostate gland and neck of the bladder; so that the mortality was far greater than was attendant on the operation of Celsus, or cutting on the gripe, as it has since been termed.

In many instances the neck of the bladder has been torn from the prostate gland, or the urethra severed from both, and so great the consequent irritation and inflammation, that death has speedily ensued. And we are informed by S.

Cooper, that "notwithstanding these serious results, this mode was practiced by Pare, Le Dran, Le Cat, Mery, Morand, Marichal, Rau, and many of the best surgeons of Europe, for several hundred years," down to the introduction, into Paris in 1697, by Frere Jaques, of the original model of the operation of lithotomy, and the invention of the lateral operation by Franco, which has undergone so many modifications and changes, both in Europe and America; and which at last seems to be the only mode by which a patient with stone can with safety and certainty be relieved.

Fabricius Hildanus, it appears, first used the round staff in lithotomy, and it was by this means he attempted to obviate the difficulties which presented themselves in the operation of Celsus. By making the staff prominent in the perineum, he cut down upon it, and with much more certainty than in the original mode; and many surgeons, even at the present day, think this a safe operation, especially in children.

Frere Jaques, adopting the staff of F. Hildanus, performed his operation by first making the convexity of the staff prominent in the perineum and plunging in the direction of the bladder, an instrument, long and dagger-like, making it enter near the tuber ischii. This singular individual appeared in Paris in 1687, and his comparatively wonderful success, at least the extravagant number of his patients, for a time attracted the attention of all Europe, and many of the most distinguished surgeons adopted his mode of operating. His instrument may be justly considered the first great step in the construction of our present gorget. The improvements of this mode of operating, by grooving the staff and putting a beak on his instrument, by the Parisian surgeons, brought this plan almost to equal the present mode of operating with the gorget. But for a long period there was no cutting edge to the poignard-like instrument, and all the openings in the bladder were made either in the form of a lacerated or punctured wound; and so great was the terror excited by the flow of blood in the minds of the older surgeons, that it was not until the days of Sir Cæsar Hawkins, that the gorget was improved by giving it a lateral cutting edge, which suggestion has proved to be far the most important that has been made in the operation, and the success attending the use of the instrument thus constructed much greater than that of any previously invented.

Since this period, many attempts at the improvement of the gorget have been made, both by the continental surgeons and those in England; but their suggestions have not added much to the facility or safety of the operation. Cheselden performed this operation with various modifications in the form of the instruments; and the present English mode of extracting the stone, both by the gorget and the knife differs but little from that so repeatedly performed, and with such unusual success, by that accomplished anatomist and surgeon.

The instrument preferred in the United States is an improvement on Cheselden's and Hawkins' gorget, by Dr. Physick. This gentleman, with his usual acuteness, discovered that much of the difficulty in the introduction of the instrument was occasioned by the want of a well-sharpened edge at the point of attachment between the beak and the cutting gorget, and his improvement consists in making the instrument so that the blade can be detached from the beak, that it may be better sharpened, and made to cut with greater ease and certainty when applied.

Such have been the various steps in the improvement of the plan of an instrument, which we think deserves the preference by the profession, over any other yet invented. And although the advancement has been but slowly made, yet it has always been in the most certain, and in by far the most successful track. Even the rude operation of Celsus, and that of Fabricius Hildanus, and Frere Jaques, are not now surpassed, as to safety and success, by many of the modern modes in the distinguished European schools.

In France, as theory after theory in pathology has been propagated, and each for a time has had its reign, so in surgery, instrument after instrument has been invented, for the operation for stone, and the bladder has been entered at every accessible point, but never with a success that has equaled the old lateral operation with the gorget.

The *lithotome cache* was for a time applauded. Frere Come has extolled his high and low operation. Dupuytren has performed various operations on the bladder, and among others the extraction of the stone through the rectum. Lisfranc, and a host of others, have proposed modes and instruments of their own, but not to the advancement of the art. Civiale has caught the stone in the bladder and bored and ground it to powder; while Heurteloup has by force broken it to pieces. But the reports and confessions of the latter gentleman rivet conviction on the mind of every intelligent surgeon, that instead of being improvements in the profession, they are but calculated to torture the patient, and greatly add to the bills of mortality.

"The beautiful apparatus of Civiale (says an English writer of high distinction) was hailed as a means of doing away entirely with any other proceeding. Some ingenious alterations were made upon it by Mr. Heurteloup and others, and at one time it was confidently asserted, that almost every patient suffering from stone could obtain a perfect and a permanent cure. Some new apparatus, (I shall not pretend to say who has the merit of the invention, for it would not be safe to interfere with the contending parties,) certainly of a more efficient kind, was introduced, and forthwith the other was, by those who had previously given an opinion, denounced as totally worthless and inefficient.

It is too true that such is the case, that you might bore holes in almost any stone without in any way advancing the patient's recovery; on the contrary, with the effect of superadding to his other maladies a thoroughly diseased bladder. This stone (he continues) was removed from the bladder, by lithotomy, in one-tenth part of the time that any of the sittings had occupied, and with certainly a fourth of the pain, and with much less danger. A very small and soft stone may be managed by this drilling apparatus, but such stones bear but a small proportion to those that are totally impracticable.

It is a matter of much astonishment that some of the ingenious gentlemen, who have busied themselves about this matter, should not have proposed introducing a charge of gunpowder into one of the perforations, and thus shattering the concretion, as rocks are blasted in the bottom of the ocean. Many people are still racking their brains, to my knowledge, to invent some apparatus superior to any used.

This is all praiseworthy, but expectations have been raised too high on this subject, by far, by unwarrantable assertions. I have practiced all the operations in a wide and extended field, and have seen others perform them, and if I might be permitted to offer an opinion on the subject, I shall say that unless the laws of the human economy are subverted, a permanent cure can not be expected to follow lithotrity unless in very favorable cases, and among patients who present themselves for relief from the pains of stone, certainly not more than one-sixth ought to be submitted to that proceeding, *and would not be* by a conscientious surgeon, who could equally as well cut out the stone as to powder it down."

An allowance should, however, be made for the hostility which exists between the French and the English surgeons. But, when all that justice would give the former is awarded, we shall find ample ground for believing, that cutting for the stone is far preferable to any other mode of treatment.

Independently of the difficulties which are so apt to arise from the growth of other stones from the fragments of stone left in the bladder, forming nuclei for after depositions, the degree of irritation that attends the introduction and long retention of the instruments for the destruction of the stone, is so great, that a large number of the patients of those who practice this mode, perish from this cause alone. They either sink during the series of operations—the nervous system being unable to sustain itself against the unceasing attacks of an adversary so formidable—or inflammation supervenes, which soon hurries the tortured victim to the grave. No discussion, however, need be entered into as to the propriety of cutting for the stone, compared with Civiale's or Heurteloup's mode of operating, since the great mass of the enlightened surgeons both of Europe and America, have facts and reasons amply sufficient to cast the balance in favor of either the gorget or the knife. This last mentioned mode of operating with the knife, (which among some of the most eminent modern surgeons has had very able advocates,) deserves much consideration, since it disputes the ground so long and justly claimed by the gorget, with more reasonable pretensions than any other method.

In the hands of a good anatomist and a judicious and careful surgeon, the knife is certainly safe; but in the hands of the inexperienced, it assuredly is a dangerous instrument.

There are, however, many objections which might be urged against the use of the knife, and we conceive that most of those which are, with great propriety, made to the gorget, as it is commonly employed, do in fact lie against the knife.

The hazard which the patient runs in the use of both, is the wounding of the rectum, or fundus of the bladder; and assuredly the one will as readily effect this injury as the other, when improperly directed. Besides, the knife in fat subjects, because of the great depth of the incision, cannot be directed with the same certainty as the gorget. It cannot be otherwise than extremely difficult for the surgeon, in *such* cases, holding the extremity of the handle of his knife, to direct it as he would desire. Thus it often happens, that the edge of the instrument is turned downwards and the vesiculæ seminales are wounded, or the neck of the bladder is mangled by the frequent attempts to enlarge the opening; and it likewise frequently occurs, that at the moment the knife has cut through the ligaments, the thickened prostate and neck of the bladder, like the gorget it will make a sudden plunge, and if not properly directed, may readily wound the bladder and rectum.

The object of this paper, however, is not to discuss at large the advantages and disadvantages of these instruments, but to offer a point for the improvement of the gorget operation.

In the study of the parts concerned in lithotomy, we were deeply impressed with the great danger to which a patient is subjected, in the performance of this operation, with the ordinary instruments. But the practice of the operation, and a knowledge of the sad results which so often follow it, directed still more our attention to the subject, and induced us to make the attempt to obviate the difficulties, and remove the obstacles in the performance of the operation with the gorget in the present mode, and render it safe and successful, even in the hands of the inexperienced surgeon. How far we have effected our design will be seen by a reference to the subjoined plate, which explains the plan of the operation, and the alledged improvements in the structure of the instrument.

### EXPLANATIONS OF THE PLATE.

*A.*—The grooved staff—which should be made of silver, because of its being less liable to be broken in the operation than the steel

*B.*—The entrance into the groove a short distance above the convexity of the staff, that the knife can with greater certainty cut down upon it.

*C.*—The extremity of the groove in the staff.

*D.*—The ball on the beak of the gorget—which should be so constructed as to screw on the beak: That when we would operate on a child, a smaller staff and blade could be employed.

*E.*—The central part of the gorget to which the blades are applied, is made of spring steel, that the beak, which is necessarily small, may not be broken by the force employed in operating

*F.*—The blades employed in the bilateral operation, and which can be removed for others, or for the purpose of operating with the single blade.

*G.*—The screw which passes through both blades.

*H.*—The handle.

*I.*—The blade detached, and made according to Physick's improvement.

Improved Gorget.

To those who are at all acquainted with the dangers of this operation, we need not offer an argument or a reason for this instrument, for its use and necessity are, at the first glance, obvious; and every operator knows how great are the difficulties attending the gorget in common use, and how much they need to be overcome. There are but few operators with this instrument, (although the best,) who, if they have used it extensively, do not have reason to complain of some accident, and would ascribe their failure to an imperfection in its formation; and who, if they spoke in candor, would not allow that some had fallen victims to its unfortunate application.

In the use of the gorget the great danger is, its slipping from the grooved staff, and injuring the surrounding parts. Thus, when the beak of the instrument is applied to the staff at an acute angle with the handle, mounting up towards the staff, if the instrument is pushed forward into the bladder in the direction originally assumed, often passing the greatest convexity of the grooved staff, it will be found to leave the staff entirely, and pass unguided into the bladder, and often into the rectum, or between the bladder and the bowel; and when the gorget is applied at an obtuse angle, it is then not so easily passed along the groove, and when too much force is applied, it has sometimes slipped upwards and forced itself between the pubis and bladder, doing great damage in that direction.

The following statements, as made by two of the most distinguished English surgeons, will suffice as proof of the dangers attending the most judicious use of this instrument.

"If I were asked," says Sir Astley Cooper, (a man whose works are characterized by honesty and candor,) "how many times I have known the gorget slip and pass between the rectum and bladder, I should say at least a dozen times, and in each case the most lamentable and fatal consequences ensued, for the operator now lays hold of the stone and the bladder together, the forceps slip, the stone enclosed in the bladder is again laid hold of, and thus he continues to pull, bruise, and injure the bladder, until the patient is taken back to his bed with the stone unextracted. Violent inflammation supervenes from the injury done to the bladder, and in a few days the patient is no more." Sir James Earle remarks, "I have more than once known a gorget, though passed in a right direction, pushed on so far and with such violence as to go through the opposite side of the bladder." And Mr. S. Cooper asserts, he has known of at least three cases in which the gorget slipped from the staff, and the urethra was completely severed from the bladder, and in all the patients died; and it is even recorded, that when the blunt gorget has been used, that such force has been employed, that the opposite wall of the bladder has been so much torn as to allow the bowels to pass down into the internal incision.

To avoid these accidents, is the design of the instrument here proposed. The staff is so constructed as to confine the beak of the gorget, from the point at which it is applied to the extremity in the bladder. If it is necessary to the safety of the patient that the beak of the gorget should traverse the grooved staff, there can be no good reason why it should not be confined in the groove.

If this be properly done, the veriest bungler may perform the operation without the slightest danger to the patient. But an objection has been urged to the gorget, that an opening sufficiently large can not be made for the passage of many stones in the bladder; for this purpose our instrument is so constructed that two blades may be applied, and the bilateral operation performed, which is far preferable, in our opinion, to cutting entirely on one side of the neck of the bladder, for the purpose of enlarging the opening. When the two blades are applied, an opening sufficiently large may be made for the extraction of a stone as large as could possibly be taken through the perinæum. But in most instances by the use of the forceps, invented by Professor Jameson, for breaking up calculii, very large openings need never be made, at all events not larger than are required for this instrument's introduction.

In the performance of lithotomy with this instrument, some have thought it would be difficult to make the incision correspond with the cut in the perinæum, but nothing can be more easily effected. Should we cut upon the staff below the opening for the reception of the ball on the beak, by which it is confined to the staff, all that is necessary is to pass the staff a little farther into the bladder, and the object is accomplished; or should we cut above, which is hardly probable, a slight withdrawel of the staff will immediately expose the place of entrance; nor are we deceived as to the certainty of its being in, as in the case of the present gorget, for so soon as we have passed it the distance of half an inch, it is confined, and cannot make its escape but by being drawn back by the operator, to the point at which it entered.

Sir Charles Blake and Le Cat are said to have devised some such instrument; but not having seen either their drawings or descriptions, I cannot tell on what plan theirs were constructed. That of Sir Charles Blake was, however, according to Mr. S. Cooper, not intended to confine or arrest the gorget at the extremity of the staff, as in the one now proposed. Thus, the dangers which have been so much dreaded could not be fully prevented, for in the plunge of the gorget into the bladder, after having passed through the thickened and often indurated prostate gland and the neck of the bladder, the rectum was as liable to injury as when the common old gorget is used, and nothing was gained by its confinement, but in its passage through the prostate, which does not constitute the danger most to be guarded against in this operation.

Mr. B. Bell, in his System of Surgery, also speaks of some invention of this kind, but objections were made to the proposed mode of operating, because of the difficulty in passing the gorget into the bladder. The instrument referred to, could not have been constructed as ours is, for not the least difficulty is found in its introduction, and the beak of the gorget is so much embraced by the staff as to bring the cutting edge so close to it as entirely to obviate the objection so much urged against the gorget—that of tearing the urethra in its introduction.

In performing lithotomy with the beak of the gorget confined, we have, also, another great advantage in avoiding the pudic artery. We are directed to incline the staff to one side when there is danger of cutting this artery, and by being too fearful of this result, we are apt to press with so much force as to dislodge it from the groove, and it is then thrust into the bladder without any guide. But when the beak is confined, we can press with as much force as is required to pass by the pudic entirely; and, beside, the staff need not be pressed with such violence against the pubis, or so high up in the arch of the bones as to bring the blade of the gorget in contact with the artery; for should we even thrust the gorget in the bladder, with the staff pressed low down in the perinæum, we cannot wound the rectum or the fundus of the bladder. The cutting gorget is prevented from going so near the end of the staff, as by any possibility to cut the bladder, for the end pushes the parieties of the organ before and from the edge of the instrument, and, notwithstanding there may be great force employed, and after the gorget has cut through the prostate and neck of the bladder, a plunge may follow, even should it be towards the fundus of the bladder, or the rectum, the staff may be carried with the cutting gorget in such way as will cause both instruments to slide on the surface of the bladder without wounding it. A trial of this instrument on the dead subject will convince any one of the facts as stated.

In suggesting these improvements to the profession, I am not at liberty to *claim* the award of originality; for although the instruments which I have had constructed, were, as I supposed, entirely of my own invention, I since find that others had entertained similar views before me. Neither their plan nor instrument, as described, could, however, accomplish what we propose and what we candidly believe, will be experienced by all who will venture the trial.

Finally, we would not say of the invention, that it renders a knowledge of the anatomy of the parts concerned unnecessary; but much less would be sufficient.

*Cincinnati, September*, 1835.

## Figures and Tables

**Figure f1:**